# Factors Related to Oral Intake of Food by Hospitalized Patients with Malnutrition under the Care of a Nutrition Support Team

**DOI:** 10.3390/ijerph182111725

**Published:** 2021-11-08

**Authors:** Junichi Furuya, Hiroyuki Suzuki, Rena Hidaka, Kazuharu Nakagawa, Kanako Yoshimi, Ayako Nakane, Kohei Yamaguchi, Yukue Shimizu, Yasuhiro Itsui, Keiko Saito, Yuji Sato, Haruka Tohara, Shunsuke Minakuchi

**Affiliations:** 1 Geriatric Dentistry, Showa University School of Dentistry, 2-1-1 Kitasenzoku, Ohta-ku, Tokyo 145-8515, Japan; furuyajunichi@gmail.com (J.F.); sato-@dent.showa-u.ac.jp (Y.S.); 2 Dysphagia Rehabilitation, Graduate School of Medical and Dental Sciences, Tokyo Medical and Dental University (TMDU), 1-5-45 Yushima, Bunkyo-ku, Tokyo 113-8549, Japan; k.nakagawa.swal@tmd.ac.jp (K.N.); k.yoshimi.gerd@tmd.ac.jp (K.Y.); a.nakane.swal@tmd.ac.jp (A.N.); yanma627@yahoo.co.jp (K.Y.); haruka-t@rd5.so-net.ne.jp (H.T.); 3 Gerodontology and Oral Rehabilitation, Graduate School of Medical and Dental Sciences, Tokyo Medical and Dental University (TMDU), 1-5-45 Yushima, Bunkyo-ku, Tokyo 113-8549, Japan; s.minakuchi.gerd@tmd.ac.jp; 4 Oral Health Sciences for Community Welfare, Graduate School of Medical and Dental Sciences, Tokyo Medical and Dental University (TMDU), 1-5-45 Yushima, Bunkyo-ku, Tokyo 113-8549, Japan; n-rena.ohcw@tmd.ac.jp; 5 Nutrition Service, Tokyo Medical and Dental University Hospital, 1-5-45 Yushima, Bunkyo-ku, Tokyo 113-8549, Japan; y.shimizu.nutr@tmd.ac.jp (Y.S.); saito-k.nutr@tmd.ac.jp (K.S.); 6 Medical Education Research and Development, Graduate School of Medical and Dental Sciences, Tokyo Medical and Dental University (TMDU), 1-5-45 Yushima, Bunkyo-ku, Tokyo 113-8519, Japan; yitsui.gast@tmd.ac.jp

**Keywords:** oral intake, malnutrition, oral health status, nutrition support team, Functional Oral Intake Scale, oral health assessment, Dysphagia Severity Scale

## Abstract

This study aimed to evaluate the role of the general condition and oral health status in determining the primary nutritional route and suitable food form for oral ingestion among malnourished inpatients. This cross-sectional study included 255 inpatients referred to a nutrition support team (NST), which included dental professionals, at an acute care hospital. We assessed the participants’ basic information, and Dysphagia Severity Scale (DSS) and Oral Health Assessment Tool (OHAT) scores. The nutritional intake mode was evaluated based on the Functional Oral Intake Scale scores at the initial NST consultation (FOIS-I), and then revised by the NST based on the participants’ general condition and oral health (FOIS-R). There was a divergence between FOIS-I and FOIS-R, with FOIS-R being significantly higher than FOIS-I (*p* < 0.001). Logistic regression analysis of FOIS-R identified that consciousness level (odds ratio (OR): 0.448; 95% confidence interval (CI): 0.214–0.935) and DSS (OR: 3.521; 95% CI: 2.574–4.815) significantly affected the oral nutrition intake. Among participants who could ingest orally (FOIS-R ≥ 3; *n* = 126), FOIS score had significant negative and positive associations with the OHAT and DSS scores, respectively. These findings suggest that appropriate assessment of oral health status, including swallowing function, might contribute to high-quality nutrition management.

## 1. Introduction

Inpatients in acute care hospitals may be malnourished due to various factors, such as decreased nutritional intake or poor digestion and absorption. These symptoms can arise from underlying diseases, difficulty in chewing and swallowing, deterioration of sensory functions such as taste and smell, or increased energy consumption associated with treatments such as surgery. In total, 13–78% of inpatients are reportedly malnourished [1]. The risk of malnutrition tends to increase with prolonged hospital stays [2], and the proportion of patients with malnutrition or at risk for malnutrition tends to increase relatively early [3,4]. The deterioration of the nutritional status occurring soon after hospital admission leads to an increased risk of complications [5] and an increased mortality rate [6]. Several inpatients in acute care hospitals are transferred to a convalescent hospital for rehabilitation after the stabilization of their disease status. Proper nutritional management is essential for their effective rehabilitation. Malnutrition in convalescent-phase rehabilitation has been shown to negatively impact the functional recovery and quality of life (QOL) of patients [7]. Therefore, providing seamless nutritional management from the acute stage is crucial. A nutrition support team (NST) manages nutrition through interprofessional collaboration among medical doctors, nurses, and dieticians and has been reported to be an effective management approach in treating inpatients with malnutrition [8,9,10]. In recent years, NSTs have been utilized in several hospitals worldwide since nutritional management is essential for improving the QOL of the patients and treating diseases [10,11].

The most appropriate mode of nutritional intake physiologically is oral ingestion, and the primary goal of the NST is to encourage oral ingestion of regular diet as much as possible. However, in cases where sufficient nutrition cannot be obtained through oral ingestion alone, or when oral ingestion is difficult due to unstable general conditions (such as the deterioration of consciousness or respiratory or circulatory conditions), enteral or intravenous feeding may be selected as the primary mode of nutritional intake [12]. In NST, multidisciplinary professionals collaborate to manage general conditions from the standpoint of nutritional support and examine the mode of proper nutrition administration. In acute care hospitals, physical invasions such as those associated with surgery can be significant, resulting in the deterioration of oral health, such as poor oral hygiene, ill-fitting dentures, and decreased chewing and swallowing function due to disturbances in consciousness or primary diseases. Such oral dysfunctions may influence the selection of the mode of nutritional intake. A recent study reported that dental interventions are associated with preventing infection [13] as well as establishing the oral intake of nutrition [14], and that dentists could play a role in improving the QOL of the patients during the perioperative period [15]. Therefore, dental professionals play a major role in NST, along with medical doctors, speech–hearing therapists, nutritionists, and other medical professionals, by evaluating the oral health status, determining the mode of nutrition intake, and adjusting the food form to maximize nutritional support.

It is not clear how oral health status affects the nutritional intake method determined by the NST for inpatients with malnutrition and requiring acute care. Therefore, we performed a cross-sectional study to clarify the role of the oral health status in determining the primary mode of nutrition, especially considering whether oral nutritional intake is possible. We also aimed to determine the ideal food form for inpatients with malnutrition as targets of NST.

## 2. Materials and Methods

### 2.1. Participants

**Figure 1:**
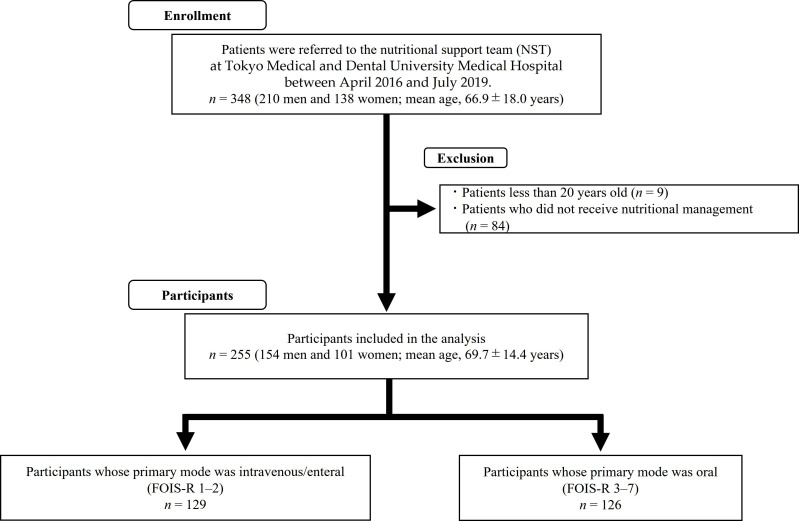
Enrollment process used in this cross-sectional study. Abbreviation: FOIS-R: Functional Oral Intake Scale-recommended.

Figure 1 shows the enrollment process used in this cross-sectional study. This cross-sectional study enrolled 348 inpatients (210 males and 138 females; mean age 66.9 ± 18.0 years) referred to the NST at Tokyo Medical and Dental University Hospital for nutritional management between April 2016 and July 2019. Patients who were less than 20 years old (*n* = 9) and those who had not received nutritional management by NST (*n* = 84) were excluded from the study. Thus, this study finally included 255 consecutive inpatients aged 20 years or older (154 males and 101 females; mean age 69.7 ± 14.4 years) with no missing data. Before starting the study, informed consent was obtained by an opt-out method. We explained the details of the study and the use of the anonymized medical information to all the participants and offered them the option to refuse participation in the study by an opt-out method. This study was approved by the ethics committee of the faculty of dentistry, Tokyo Medical and Dental University (approval number: D2016-077).

### 2.2. Outcomes

#### 2.2.1. Mode of Nutritional Intake

The mode of nutritional intake of the participants was evaluated using a seven-point scale based on the Functional Oral Intake Scale (FOIS) [16]. A previous study reported some discrepancy between the actual and appropriate mode of nutritional intake based on the general condition and oral function of inpatients in acute care hospitals [17]. Therefore, we evaluated two types of nutrition intake modes among the participants. The first was the nutritional intake mode at the initial NST consultation, which was decided by the attending medical doctor without oral health assessment (FOIS-I) before NST consultation. The other was the recommended mode of nutrition intake (FOIS-R). FOIS-R was revised after consultation among NST members, including medical doctors, dental professionals, nurses, speech pathologists, and nutritionists, based on a comprehensive evaluation of the patient’s general condition and an assessment of oral health, including the swallowing ability.

#### 2.2.2. Oral Outcomes

The oral outcomes were assessed by two well-trained dentists who performed the assessments based on standardized criteria. The swallowing ability based on the Dysphagia Severity Scale (DSS) [18] and comprehensive assessment of the oral health using Oral Health Assessment Tool (OHAT) score [19] were assessed as the oral outcomes. The DSS is a comprehensive evaluation tool used to assess the severity of dysphagia using a seven-scale rating. A lower score was associated with greater severity of dysphagia. OHAT is a comprehensive assessment tool for oral cavities, involving the evaluation of the lips, tongue, gingiva/mucous membrane, saliva, remaining teeth, dentures, oral hygiene, and toothache on a three-scale rating from 0 to 2. A higher score was associated with poor oral health.

#### 2.2.3. Other Variables

In addition, basic participant information at the initial NST consultation (age, sex, body mass index (BMI), serum albumin levels (Alb), serum C-reactive protein (CRP) levels, systemic diseases, number of days from admission until initial NST consultation, and consciousness level) was extracted from the medical records. Systemic diseases, including primary diseases and comorbidities, were scored using the Charlson Comorbidity Index (CCI) [20]. The consciousness level was recorded as one of the following three levels according to the Japan Coma Scale (JCS) [21]: “(I) alert and conscious”, “(II) awake when stimulated”, and “(III) not awake with stimulus”.

### 2.3. Statistical Analysis

The divergence between FOIS-I and FOIS-R was compared using the Wilcoxon signed-rank test. The study participants were divided into two groups according to the primary recommended nutritional route: one with an FOIS-R score of 1 or 2 (the primary route was intravenous/enteral nutrition) and the other with an FOIS-R score in the range of 3–7 (the primary route was oral nutrition). Basic participant information and oral health were compared between the groups. We performed a χ^2^ test to compare the sex and consciousness level differences, and a Mann–Whitney U test for the other items. We also performed a logistic regression analysis to examine the factors affecting oral intake. The objective variable was whether oral intake was the primary nutrition route (FOIS-R 1–2 = 0; FOIS-R 3–7 = 1), and the explanatory variables were age, sex (male = 0; female = 1), BMI, Alb, CRP, CCI score, number of days from hospital admission to initial NST consultation, JCS level, the total score of OHAT, and DSS score. Further, multiple regression analysis was performed with FOIS-R as an objective variable and age, sex (male = 0; female = 1), BMI, Alb, CRP, CCI score, number of days from hospital admission to initial NST consultation, JCS level, OHAT, and DSS scores as explanatory variables in the participants whose primary mode of nutrition intake was oral (FOIS-R 3–7) to examine the factors influencing the food form. Statistical analysis was performed using SPSS Statistics Ver. 26.0 (SPSS Institute Inc., Chicago, IL, USA), and the significance level in all statistical analyses was set at 5%.

## 3. Results

### 3.1. Divergence between the Nutritional Intake Mode at the Initial NST Consultation and the Recommended Mode of Intake

Table 1 shows the distribution of the nutritional intake mode at the initial NST consultation (FOIS-I) and the recommended nutritional intake mode based on the general condition and oral health status (FOIS-R) of the participants. Significant divergence was seen between FOIS-I (mean: 3.1 ± 2.4; median: 2) and FOIS-R (mean: 3.4 ± 2.4; median: 2) (*p* < 0.001). Divergence was seen in 36.8% of the participants, and a nutrition intake method of a lower level than the recommended method was seen in 27.8% of the participants. Conversely, a nutrition intake method of a higher level than their function existed in 9% of the participants. The results showed that at the initial NST consultation, about 50% of the participants were unable to take nutrition orally, and enteral or intravenous feeding alone was used. However, NST evaluation of the appropriate mode of nutrition intake revealed that the percentage of participants requiring nutrition intake only by enteral or intravenous feeding was 40% or less.

### 3.2. Factors Affecting Primary Nutritional Intake Modes

The participants were divided into two groups according to the primary recommended mode of nutritional intake: the group whose primary mode was intravenous/enteral (FOIS-R 1–2 group; *n* = 129), and the group whose primary mode was oral (FOIS-R 3–7 group; *n* = 126). Results of the comparison of the basic information and oral health status of the patients between the two groups are shown in Table 2. A significant difference in age (*p* = 0.048), number of days from hospital admission until the initial NST consultation (*p* = 0.005), and consciousness level (*p* <0.001) was found between the two groups. A significant difference was observed between the two groups with respect to DSS scores (*p* < 0.001), total OHAT scores (*p* = 0.027), and the components of OHAT including lip (*p* = 0.012), tongue (*p* = 0.001), saliva (*p* < 0.001), and oral pain (*p* = 0.033). These results indicated that the FOIS-R 3–7 group had a better oral health status than the FOIS 1–2 group.

Table 3 shows the results of logistic regression analysis, using the primary recommended mode of nutritional intake as the objective variable (FOIS-R 1–2: 0; FOIS-R 3–7: 1). The consciousness level evaluated using JCS (odds ratio (OR): 0.448; 95% confidence interval (CI): 0.214–0.935) and swallowing function evaluated using DSS (OR: 3.521; 95% CI: 2.574–4.815) significantly influenced whether the primary nutrition intake mode would be oral.

### 3.3. Factors Affecting Food Form Determination with Oral Ingestion as the Primary Nutritional Intake Mode

A multiple regression analysis was performed to elucidate the factors involved in food form determination in participants utilizing NST, where the primary recommended mode of nutritional intake is oral ingestion, with FOIS-R scores as the objective variable in participants with FOIS-R 3–7 (*n* = 126) (Table 4). The results showed that the oral health status (assessed by OHAT scores) and swallowing function (assessed by the DSS) significantly influenced the food form decision in participants obtaining nutrition mainly by oral ingestion.

## 4. Discussion

The findings of this study revealed a divergence between the nutritional intake route that the patients received at the initial NST consultation and the recommended mode based on the evaluation of the general condition and oral health of the patients by the NST at an acute care hospital. A lower level of nutritional intake method than the actual oral intake ability had been selected for most patients. It was also suggested that selecting oral intake as the primary mode of nutritional intake required good swallowing function and no consciousness disturbance. Moreover, the food form must be improved for efficient nourishment and QOL to promote oral ingestion. In addition to the swallowing function, good oral health status is important in improving the food form. The evaluation of appropriate nutritional intake is indispensable for patients utilizing NST in the acute phase of hospitalization. The findings of this study suggested that the participation of dental professionals and the appropriate assessment of oral health by them may prevent unnecessary fasting and contribute to high-quality oral nutrition intake.

The restriction of oral intake decreases the QOL of inpatients and causes a decline in the swallowing function due to disuse, including muscle atrophy [22]. In addition, it has been reported that those who fasted temporarily during hospitalization required a longer treatment period and had a decline in their swallowing function compared to those who continued oral intake [23], suggesting that it is important to maintain oral intake even in inpatients in the acute phase of hospitalization. However, enteral or intravenous feeding should be utilized in inpatients with an unstable general condition to ensure nutritional management [12,24], improve nutritional status, and prevent complications [25]. It has been reported that the disuse of the intestine for a prolonged period causes intestinal mucosal atrophy and negatively affects the intestinal immune system, resulting in a higher risk of infection and multiple organ failure [26]. Therefore, considering a mode of nutritional intake that involves intestinal use as early as possible is recommended [27]. The nutritional intake mode appropriate for each patient differs. An essential expected role of the NST is to accurately evaluate the general condition and oral health status of each patient to determine the optimal mode of nutritional intake [11]. Divergence between the two nutrition intake modes, especially the actual nutritional intake mode being lower than the recommended nutritional intake mode, suggested improper evaluation of the necessary mode of nutrition intake among inpatients during the acute phase of hospitalization. It might also reinforce the significance of NST, including dental professionals in acute care hospitals.

When comparing the FOIS-R 1–2 group with the FOIS-R 3–7 group, the FOIS-R 1–2 group had a significantly lower swallowing ability and had more patients with lip, tongue, and xerostomia problems than the FOIS-R 3–7 group. These results were similar to those reported in recent studies on oral health in patients with dysphagia [17,28].

In terms of examining the mode of nutritional intake, NST first determined whether oral intake is safe. The risk of aspiration pneumonia can increase when oral intake is impossible [29]. The short and long-term prognosis of aspiration pneumonia is poor and is associated with prolongation of the hospital stay and increased mortality [30]. In addition to the activities of daily living and level of consciousness, oral functions such as swallowing and chewing affect the selection of the nutritional intake method for inpatients in acute phase hospitals [17]. Clinicians refer to the consciousness level, respiratory condition, and swallowing function to determine the timing of restarting oral intake in patients with aspiration pneumonia [30]. In this study of NST patients, as reported in a previous study of patients with aspiration pneumonia [31], the level of consciousness and swallowing function affected the determination of the primary nutritional intake mode. This indicated that maintaining a favorable consciousness level and swallowing function is essential for safe oral intake.

If oral intake is possible, it is desirable to ingest food in a form as close to normal as possible. Modified food provided for impaired swallowing involves the use of thickened fluids containing more water than regular meals, resulting in a lower calorie intake upon ingesting the same weight of food. Furthermore, chewing is necessary when the food form is relatively solid. Chewing movements promote bowel motility [32], and the chewed food is simultaneously crushed and mixed with saliva, promoting digestion and absorption through the salivary digestive enzymes [33]. In addition, eating a normal meal by chewing may lead to an improvement in QOL [34,35,36]. In this study, the swallowing function and oral health affected the food form being administered to the participants. This result suggested that comprehensive dental care and oral hygiene care were important to provide a higher level of food form in malnourished patients utilizing NST.

This study has several limitations. The NST in this study was of a consultation type, and its service was provided only upon the request of the attending medical doctor and did not cover all malnourished patients in the hospital. In addition, enteral nutrition could not be utilized in some patients due to intestinal diseases, and intravenous nutrition was required despite the swallowing function being normal [37,38]. These patients were also included in our analysis to model the entirety of patients utilizing NST. Moreover, in this study, the systemic diseases that caused hospitalization were assessed using CCI. However, their severity or the cause of hospitalization (e.g., for surgery) were not assessed. Since nutritional intake methods may vary depending on the severity of the disease and the magnitude of the invasion, the lack of assessment of these factors may have affected the accuracy of evaluation of the relationship between nutritional intake and oral health. Lastly, the appetite and caloric intake were not assessed in this study, since the eating rate was not evaluated. It is known that appetite tends to decrease in hospitalized patients [39], and nutritional intake methods are related to appetite and caloric intake [40]. If the appetite decreases, the caloric intake is also likely to decrease [41]. The assessment of the appetite and caloric intake of the participants in this study could have clarified the relationship between oral health and appetite in hospitalized patients and effective dental interventions to achieve necessary and sufficient caloric intake. The results of this study indicated a relationship between nutritional intake and oral health. Therefore, the active participation of dentists in NST and the implementation of oral health management may lead to the improvement of the nutritional intake of NST patients. However, as this was a cross-sectional study, it is difficult to prove a causal relationship. Therefore, we believe that interventional studies are required in the future to investigate the effect of active dental intervention on the nutritional intake of NST patients.

## 5. Conclusions

The results of this study revealed a divergence between the actual nutrition intake method without oral health assessment and the recommended nutritional intake methods after oral health assessment in NST patients in acute care hospitals. Most of the patients who had divergence between the actual and the recommended nutritional method were selected for lower nutritional intake methods than the recommended nutritional intake method. Moreover, oral health, including swallowing function, and the general condition of the patients were associated with recommended nutritional intake mode. These findings suggest that the appropriate assessment of the oral health status by dental professionals is important for NST at an acute care hospital and might contribute to high-quality nutrition management.

## Figures and Tables

**Table 1:** Patient distribution of each FOIS score at the initial nutrition support team consultation (FOIS-I) and the recommended mode of nutritional intake (FOIS-R).

FOIS	FOIS-I		FOIS-R *	
	*n*	%	*n*	%
1	123	48.3	95	37.3
2	25	9.8	34	13.3
3	9	3.5	9	3.5
4	10	3.9	13	5.1
5	21	8.2	29	11.4
6	36	14.1	40	15.7
7	31	12.2	35	13.7

Abbreviations: FOIS: Functional Oral Intake Scale. * *p* < 0.001, FOIS-I vs. FOIS-R; Wilcoxon signed-rank test.

**Table 2:** Comparison of participants’ basic information and oral outcomes between the intravenous/enteral nutrition group (FOIS-R of 1–2) and the oral intake group (FOIS-R of 3–7).

		FOIS-R 1–2 Group				FOIS-R 3–7 Group				
		Mean ± SD	Median	*n*	%	Mean ± SD	Median	*n*	%	*p*-Value
Age		71.4 ± 13.6	74	129		68.0 ± 15.0	71	126		0.048 *
Sex	Male			80	62.0			74	58.7	0.592
	Female			49	38.0			52	41.3	
BMI (kg/m^2^)		20.5 ± 4.4	20.2	129		20.3 ± 4.8	20.1	126		0.63
Alb		2.4 ± 0.6	2.4	129		2.5 ± 0.6	2.4	126		0.567
CRP		6.0 ± 6.9	3.1	129		5.6 ± 6.0	4.1	126		0.808
CCI score		2.4 ± 2.2	2	129		2.6 ± 2.4	2	126		0.752
Days from hospital admission until NST consultation		33.0 ± 38.2	21	129		24.7 ± 42.2	14	126		0.005 *
JCS level	0			20	15.5			61	48.4	<0.001 **
	I			70	54.3			63	50.0	
	II			20	15.5			0	0.0	
	III			19	14.7			2	1.6	
DSS score		2.2 ± 1.5	2	129		5.3 ± 1.2	5	126		<0.001 *
	1			56	43.4			0	0.0	
	2			37	28.7			1	0.8	
	3			15	11.6			8	6.3	
	4			10	7.8			29	23.0	
	5			4	3.1			29	23.0	
	6			4	3.1			38	30.2	
	7			3	2.3			21	16.7	
OHAT total score		5.1 ± 3.2	4	129		4.3 ± 2.9	4	126		0.027 *
OHAT score	Lips	0.5 ± 0.6	0			0.4 ± 0.6	0			0.012 *
	Tongue	0.8 ± 0.7	1			0.5 ± 0.6	0			0.001 *
	Gum/mucosa	0.5 ± 0.7	0			0.5 ± 0.6	0			0.783
	Saliva	1.0 ± 0.7	1			0.6 ± 0.6	1			<0.001 *
	Teeth	0.5 ± 0.7	0			0.5 ± 0.8	0			0.89
	Dentures	1.0 ± 1.0	1			0.9 ± 1.0	0			0.244
	Oral cleanness	0.8 ± 0.8	1			0.7 ± 0.7	0.5			0.186
	Dental pain	0.2 ± 0.5	0			0.3 ± 0.6	0			0.033 *

Abbreviations: FOIS-R: Functional Oral Intake Scale-recommended; SD: standard deviation; BMI: body mass index; Alb: serum albumin; CRP: serum C-reactive protein; CCI: Charlson Comorbidity Index; NST: nutritional support team; JCS: Japan Coma Scale; OHAT: Oral Health Assessment Tool; DSS, Dysphagia Severity Scale. * *p* < 0.05 FOIS 1–2 vs. FOIS 3–7; Mann–Whitney U-test. ** *p* < 0.05 FOIS 1–2 vs. FOIS 3–7; chi-square test.

**Table 3:** Logistic regression analysis with oral intake as the primary nutrition route being the objective variable (FOIS-R 1–2 = 0; FOIS 3–7 = 1) (*n* = 255).

Independent Variables	Odds Ratio	95% Confidence Interval	*p*-Value
Age	1.029	0.996 to 1.062	0.082
Sex ^a^	0.840	0.360 to 1.959	0.687
BMI	0.995	0.909 to 1.089	0.908
Alb	0.731	0.359 to 1.488	0.387
CRP	1.011	0.950 to 1.076	0.723
CCI	1.003	0.843 to 1.193	0.972
Days from hospital admission until NST intervention	0.996	0.983 to 1.010	0.577
JCS	0.448	0.214 to 0.935	0.032 *
OHAT	1.007	0.876 to 1.157	0.923
DSS	3.521	2.574 to 4.815	<0.001 *

Abbreviations: FOIS-R: Functional Oral Intake Scale-recommended; BMI: body mass index; Alb: serum albumin; CRP: serum C-reactive protein; CCI: Charlson Comorbidity Index; NST: nutritional support team; JCS: Japan Coma Scale; OHAT: Oral Health Assessment Tool; DSS: Dysphagia Severity Scale. ^a^ Sex: male = 0, female = 1; The other independent variables were used as continuous variables. * *p* < 0.05, logistic regression analysis.

**Table 4:** Multiple regression analysis using FOIS-R as the objective variable in participants with FOIS-R 3–7 (*n* = 126).

Independent Variables	B ^a^	SE ^b^	95% Confidence Interval	β ^c^	*p*-Value	Variance Inflation Factor
Age	0.004	0.006	−0.009 to 0.016	0.044	0.563	1.337
Sex ^d^	−0.005	0.171	−0.344 to 0.333	−0.002	0.975	1.138
BMI	0.025	0.017	−0.010 to −0.059	0.098	0.156	1.088
Alb	−0.023	0.144	−0.308 to −0.262	−0.012	0.873	1.233
CRP	0.027	0.015	−0.001 to −0.056	0.137	0.063	1.221
CCI	−0.009	0.036	−0.079 to 0.062	−0.017	0.810	1.179
Days from hospital admission until NST intervention	−0.001	0.002	−0.005 to 0.003	−0.038	0.580	1.078
JCS	−0.033	0.143	−0.316 to 0.250	−0.016	0.817	1.126
OHAT	−0.073	0.029	−0.132 to −0.015	−0.181	0.014 *	1.200
DSS	0.623	0.074	0.477 to 0.768	0.630	<0.001 *	1.270

Abbreviations: FOIS-R: Functional Oral Intake Scale-recommended; BMI: body mass index; Alb: serum albumin; CRP: serum C-reactive protein; CCI: Charlson Comorbidity Index; NST: nutritional support team; JCS: Japan Coma Scale; OHAT: Oral Health Assessment Tool; DSS: Dysphagia Severity Scale. Multiple R = 0.705; R^2^ = 0.497; *p* < 0.001. ^a^ B: partial regression coefficient; ^b^ SE: standard error; ^c^ β: standardized partial regression coefficient; ^d^ Sex: male = 0, female = 1. All other independent variables were used as continuous variables. * *p* < 0.05, multiple regression analysis.

## Data Availability

Not applicable.

## Abbreviations

Alb, serum albumin; BMI, body mass index; CCI, Charlson Comorbidity Index; CRP, serum C-reactive protein; DSS, Dysphagia Severity Scale; FOIS, Functional Oral Intake Scale; FOIS-I, Functional Oral Intake Scale-initial; FOIS-R, Functional Oral Intake Scale-recommended; JCS, Japan Coma Scale; NST, nutritional support team; OHAT, Oral Health Assessment Tool.
